# Experimental Study of the Deposition of Magnetic Particles on the Walls of Microchannels

**DOI:** 10.3390/mi12060712

**Published:** 2021-06-17

**Authors:** Sylvana Varela, Antonio Rivas, Anton Vernet, Jordi Pallarès

**Affiliations:** Department of Mechanical Engineering, Universitat Rovira i Virgili, 43007 Tarragona, Spain; sylvana.varela@urv.cat (S.V.); antoniojose.rivas@urv.cat (A.R.); anton.vernet@urv.cat (A.V.)

**Keywords:** magnetic beads, microchannel, particle deposition

## Abstract

This study analyzes experimentally the deposition of magnetic beads on the walls of a square microchannel by the action of a nearby cubical magnet. The deposition has been studied for different magnetic bead sizes, flow rates, magnetic conditions and with solutions of magnetic and non-magnetic particles. Images of the time evolution of the deposition under the different conditions have been analyzed to determine the spatial distribution of the accumulation and the growth rate of the depositions. It has been found that the way in which the magnetic beads are deposited on the walls of the microchannel depends strongly on their size and the magnetic configuration. The accumulation of the major part of particles is on the wall closest to the magnet and, depending on the size of the particles, near the magnet leading and trailing edges or near the center of the magnet. The experiments with magnetic and non-magnetic particles revealed the screening effect of the non-magnetic particles on the deposition. In this case, the non-magnetic particles displace the deposition toward the region near the center of the magnet and near the trailing edge.

## 1. Introduction

Nowadays miniaturized lab-on-a-chip devices have become powerful tools to analyze, manipulate, and control droplets [1,2,3,4,5], biological particles [6,7,8], and active colloids [9,10,11].

In lab-on-a-chip devices, magnetic forces are commonly used to manipulate the position of microscopic particles [12,13,14] or artificial microswimmers [15,16].

Magnetic beads are used mostly for in vitro applications (bio-diagnostics and bio-recognition) and recently for in vivo applications, such as cancer treatment. In this case, functionalized magnetic particles can be transported by the blood flow and retained by a magnet implemented in the treatment zone, avoiding more aggressive treatments like X-ray proton therapy or chemotherapy which may produce side effects. For the most common applications, the sizes of the magnetic particles range from 5 nm to 6 μm [17].

Zhou et al. [18] showed that paramagnetic ellipsoidal particles could be focused to the channel center by applying a static uniform magnetic field perpendicular to the flow.

The introduction of non-magnetic particles in the system has implications on in vitro applications [19,20,21], such as bio-diagnostics and bio-recognition [22,23,24,25,26], and in the interaction between erythrocytes and thrombocytes [27]. Specifically, in our study the experiments have been conducted with particle sizes similar to the particle diameters found in blood. The application of the magnet near the wall of the microchannel produces the attraction of the magnetic particles, which can simulate intravenous treatments (e.g., in vivo application [28,29] such as cancer treatment [30]) or the behavior of erythrocytes and thrombocytes in the early stages of the thrombosis [31,32,33] when the wall of a blood vessel is injured.

In the present work, we explore the different forms of deposition of spherical magnetic particles in a constant magnetic field generated by a permanent magnet. We consider different orientations of the magnetic field, different sizes of particles, and different flow rates. One experimental condition has been chosen to include non-magnetic particles. In this case, the measurements have been carried out adding and modifying the concentration of non-magnetic particles to determine how they influence the quantity and shape of the deposition.

## 2. Materials and Methods

**Figure 1:**
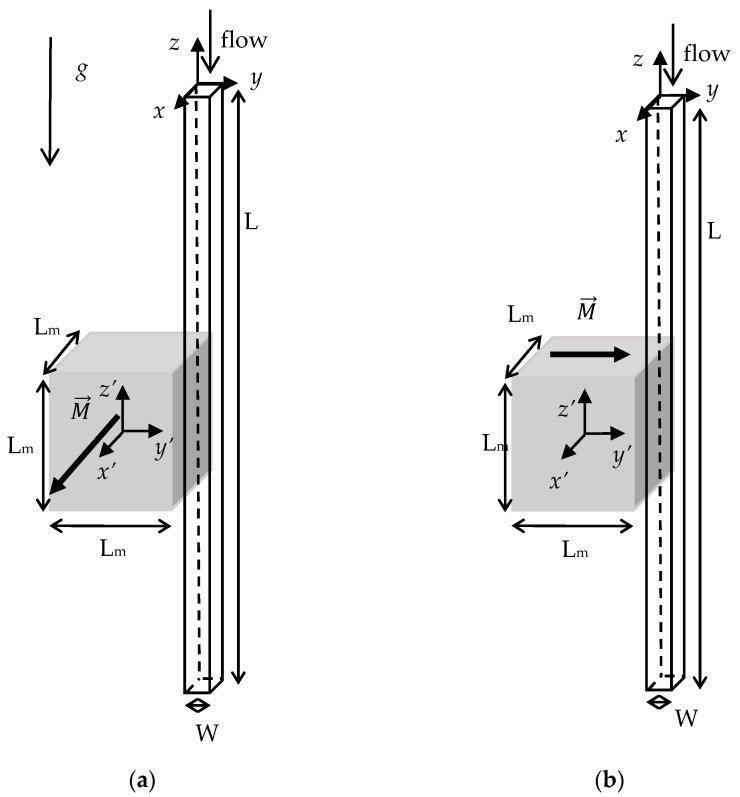
Physical model showing the position of the magnet and the orientation of the magnetization vector with respect to the flow direction. (**a**) Parallel (PA), (**b**) perpendicular (PE). Gravity acts along the negative z direction.

A permanent cubical magnet is located on the side of a straight microchannel with a square cross section. Two different orientations of the magnet have been considered, as shown in Figure 1.

In Figure 1a the magnetization vector is parallel to the side of the magnet which is closest to the wall of the microchannel ($\vec{M}=M\;\hat{x\prime }$) and Figure 1b shows the perpendicular position, in which the magnetization vector is perpendicular to the side of the magnet closest to the microchannel ($\vec{M}=M\;\hat{y\prime }$). Gravity vector is in the negative *z* direction, parallel to the main flow direction, to avoid gravitational settling of the particles on the walls of the microchannel.

The fluid used was deionized water and the magnetic particles were carboxyl spherical particles with mean diameter of 1.14 and 4.37 µm purchased from Spherotech, Inc. [34]. Some experiments were carried out with suspensions of magnetic particles and some other experiments with suspensions of magnetic and non-magnetic particles. The experiments with only magnetic particles were performed with solutions with concentrations of approximately 8.16 × 10^5^ particles/µL and 1.45 × 10^4^ particles/µL, for the smaller and larger particles, respectively. For both magnetic particle sizes, the corresponding volume fraction in the solutions used for the experiments carried out (with only magnetic particles) is 6.33 × 10^−4^.

The experiments with magnetic and non-magnetic particles were carried out with magnetic particles of 1.14 μm and hollow glass spheres [35] of 10 μm of diameter.

Experiments with magnetic and non-magnetic particles were performed with a constant concentration of magnetic particles (8.16 × 10^5^ particles/µL) and four different concentrations of non-magnetic particles with values 1.74 × 10^3^ particles/µL, 3.47 × 10^3^ particles/µL, 6.95 × 10^3^ particles/µL, and 1.04 × 10^4^ particles/µL. In these solutions, the volume fraction of magnetic particles was kept constant and the corresponding volume fractions of non-magnetic particles were 9.10 × 10^−4^, 1.82 × 10^−3^, 3.64 × 10^−3^, and 5.46 × 10^−3^.

The magnets used were Neodymium (NdFeB) cubical magnets with sizes of 3 and 5 mm and a residual magnetic field (*B*_r_) of 1.32–1.37 T and 1.29–1.32 T, respectively [36]. The magnet was installed, as shown in Figure 1, near transparent square capillaries manufactured in borosilicate glass (Vitrocom Inc., Mountain Lakes, NJ, USA [37]). Two different sizes of microchannels were used with hydraulic diameters of 300 µm and 600 µm. The length of the microchannels was 100 mm.

**Figure 2:**
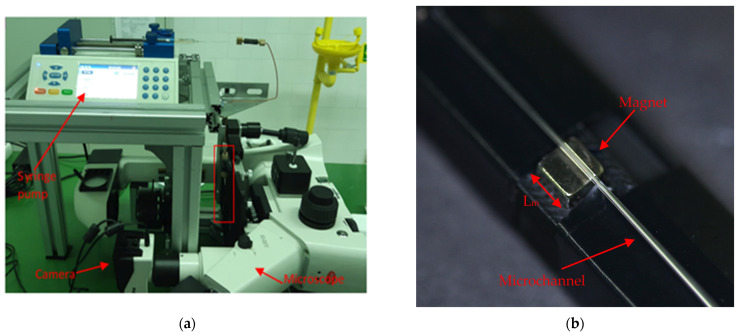
Experimental setup. (**a**) General view. The red rectangle indicates the zone shown in (**b**) with the cubical magnet and the microchannel.

An epifluorescence microscope (Motic AE31E TRI) was connected to a camera (Mo-ticam Pro 285B, 1.4 Mp, maximum frame rate 15 fps) to visualize and record the images of the time evolution of the particle deposition near the wall adjacent to the magnet. The flow was supplied with a syringe pump (Fusion 710) which ensured a constant flow rate during the experiments, which typically lasted between 8 and 33 min. The typical experimental arrangement is shown in Figure 2.

The flow is assumed incompressible, steady, and laminar. The maximum Reynolds number for the experimental conditions is 6.47.

For each experiment, the time evolution of the deposition was recorded with 999 images. The time between images was adjusted to 2, 1, or 0.5 s for flow rates of 0.03, 0.06, or 0.12 mL/min, respectively. Thus, the image 999 corresponds to the exact time the syringe of the pump was fully emptied.

**Figure 3:**
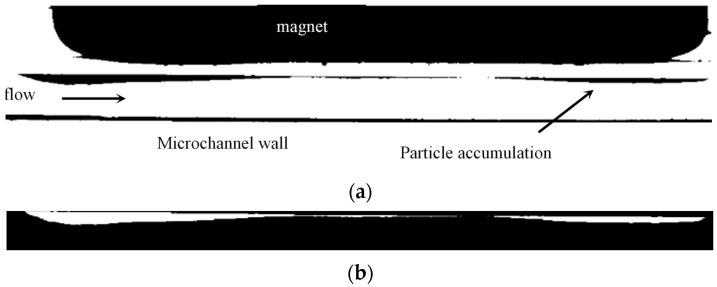
Example of a recorded and a processed image (**a**) before mask subtracting with the particle deposit in black and (**b**) after mask subtracting with the particle deposit in white.

A mask obtained from an initial image without any deposition of particles was subtracted from all the binarized recorded images. An example of a binarized image before and after the application of the mask is shown in Figure 3. In this way we can calculate in each temporal step the area of the projection of the accumulated particles on the plane yz and their shape using Matlab functions. It can be seen that the deposition can be clearly identified in the processed image.

## 3. Results

A total of 16 experiments were performed. Table 1 summarizes the different experimental conditions used. The experiments were grouped into six sets and were labelled according to the following nomenclature:

- PA or PE indicate the parallel or the perpendicular direction of the magnetization vector, respectively (see Figure 1).
- M3 or M5 indicate the size of the magnet (3 or 5 mm).
- P1 or P4, the size of the magnetic particles.
- C3 or C6 the hydraulic diameter of the microchannel (300 or 600 μm).
- QL, QM, and QH, the low, medium or high flow rate.
- PNM indicates the parts per ten thousands volume fraction of non-magnetic particles in the solution (09, 18, 36, or 55).

The first and second experimental sets were oriented toward the study of the effect of the flow rate and the orientation of the magnetization vector. In the third, fourth, and fifth sets the effects of the size of the magnet, the diameter of the particles, and the diameter of the microchannel were analyzed. Finally, in the sixth set the effect of the concentration of non-magnetic particles on the deposition of the magnetic beads was investigated.

In the following subsections, we analyze separately the different effects indicated in Table 1 on the rate of deposition of the magnetic particles and on the shape of the deposition.

### 3.1. Effect the Orientation of the Magnetization Vector and of the Flow Rate

**Figure 4:**
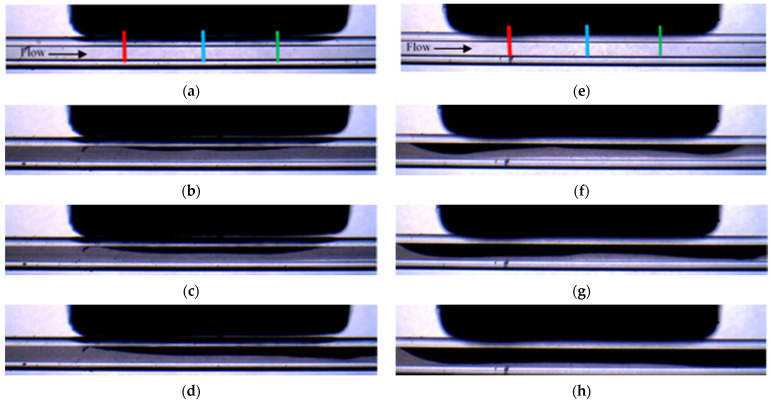
Snapshots of the deposition for experiment PA-M5-P1-C3-QL (**a**–**d**) and for PE-M5-P1-C3-QL (**e**–**h**). The non-dimensional time are for (**a**,**e**) $t{\;}^{*}$ = 4.47 × 10^1^, for (**b**,**f**) $t{\;}^{*}$ = 4.47 × 10^3^, for (**c**,**g**) $t{\;}^{*}$ = 8.95 × 10^3^ and for (**d**,**h**) $t^{\;*}$ = 1.79 × 10^4^. The lines in (**a**,**e**) indicate the positions where the accumulation is monitored, ${z^{\prime }}_{\frac{1}{4}}$ (red line), ${z^{\prime }}_{0}$ (blue line) and ${z^{\prime }}_{-\frac{1}{4}}$ (green line).

Figure 4 shows the selected snapshots of the depositions obtained for experiments PA-M5-P1-C3-QL and PE-M5-P1-C3-QL carried out with the same conditions but with different orientation of the magnetization vector. The experiment with the parallel orientation (see Figure 1a) is shown in Figure 4a–d, while the experiment with the perpendicular orientation (see Figure 1b) is in Figure 4e–h. The images of the two experiments correspond to the same non-dimensional time defined with the averaged flow velocity ($\bar{V}$) and the hydraulic diameter of the microchannel ($t^{*}=\Delta t\;.\;nframes.\;\bar{V}/W$). It can be seen in Figure 4 that for the parallel orientation, the accumulation of particles starts in the central part of the magnet, and then progressively is enlarged toward the edges of the magnet. The deposition corresponding to the perpendicular orientation starts near the edges of the magnet and is progressively enlarged near the central part of the magnet. The comparison of the depositions in Figure 4b,f shows that the perpendicular orientation generates faster deposition rates. These different behaviors agree with the larger gradients of the magnetic field, which is proportional to the magnetic force, generated within the fluid by the perpendicular orientation near the edges of the magnet.

To monitor the spatial distribution of the particles, the time of evolution of the thickness of the deposition along the y direction was measured. Three positions, relative to the coordinate system $\left(x^{\prime },\;y^{\prime },z^{\prime }\right)$ with origin in the center of the magnet (see Figure 1) have been selected. These positions were located near the leading edge of the magnet at $y^{\prime }=\frac{L_{m}}{2},\;{z^{\prime }}_{\frac{1}{4}}=\frac{L_{m}}{4}$ at the center of the magnet $y^{\prime }=\frac{L_{m}}{2},\;{z^{\prime }}_{0}=0$, and near the trailing edge of the magnet $y^{\prime }=\frac{L_{m}}{2},{z^{\prime }}_{-\frac{1}{4}}=-\frac{L_{m}}{4}.$ It should be noted that the measurements correspond to the projection of the accumulation of particles along the x direction; lines red, blue, and green respectively. For simplicity, these positions are named as ${z^{\prime }}_{\frac{1}{4}}$ (red line), ${z^{\prime }}_{0}$ (blue line) and ${z^{\prime }}_{-\frac{1}{4}}$ (green line) (see Figure 4a,e) in the following sections.

**Figure 5:**
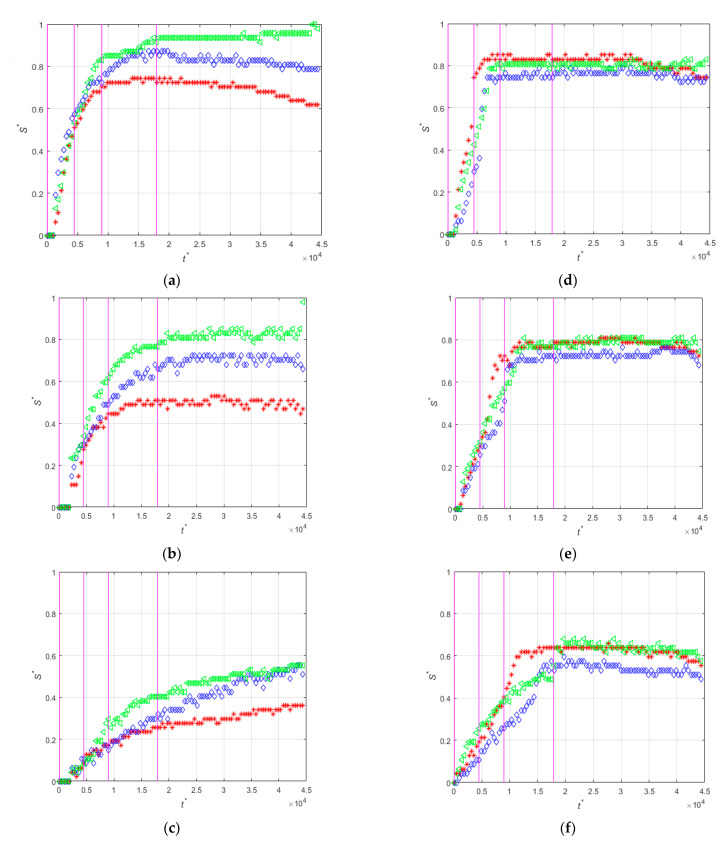
Time-evolutions of the non-dimensional thickness of the magnetic particle deposits at positions ${z^{\prime }}_{\frac{1}{4}}$ (red),$\;{z^{\prime }}_{0}$ (blue), and ${z^{\prime }}_{-\frac{1}{4}}$ (green). Low flow rate, (**a**) PA-M5-P1-C3-QL and (**d**) PE-M5-P1-C3-QL. Medium flow rate (**b**) PA-M5-P1-C3-QM and (**e**) PE-M5-P1-C3-QM. High flow rate, (**c**) PA-M5-P1-C3-QH and (**f**) PE-M5-P1-C3-QH. Vertical lines indicate times $t^{*}$ = 4.47 × 10^1^, $t^{*}$ = 4.47 × 10^3^, $t^{*}$ = 8.95 × 10^3^, and $t^{*}$ = 1.79 × 10^4^ which correspond to the snapshots of Figure 4.

Figure 5 shows the time evolutions of the non-dimensional thickness ($S^{*}=S/W$) of the magnetic particles deposited in the three different positions of the magnet indicated in Figure 4a, for the three flow rates and for the two directions of the magnetization vector.

It is evident that for the two orientations of the magnetization vector the increase of the flow rate decreases the particle deposition rate. For example for the perpendicular orientation (Figure 5d–f) the thicknesses of the particle deposit reach a plateau at non-dimensional times of approximately $t{\;}^{*}$ = 0.8 × 10^4^, 1.2 × 10^4^, and 1.7 × 10^4^, for the low, medium, and high flow rates, respectively. Note that the dimensionless time used in the plots is defined using the average fluid velocity. When the magnetization vector is parallel to the microchannel wall, the particles start to accumulate near the center of the magnet and then around the leading and trailing edges for all flow rates, Figure 5a–c shows this. For this orientation of the magnetization vector and at the lowest flow rate, the thicknesses of the deposits near the leading edge (red curve in Figure 5a) and near the center (blue curve in Figure 5a) of the magnet decrease for $t{\;}^{*}$ > 1.5 × 10^4^ indicating that the deposited particles are dragged by the action of the fluid velocity toward the trailing edge (green curve in Figure 5a) of the magnet because the relatively small magnetization force. This phenomenon is not observed for the perpendicular orientation of the magnetization vector that generates a much stronger magnetic force on the particles.

### 3.2. Effect of the Size of the Magnet

The size of the magnet directly influences the magnitude of the magnetic flux density (B) generated and, consequently, affects the force experienced by a magnetic particle, which is proportional to the vector·$\left(B\cdot \nabla \right)B$ [38]. In general, for magnets with the same or with similar magnetization, the increase of the size of the magnet increases the magnetic force toward the magnet near the edges. There is also a reduction in the central part of the magnet. The experiments for Set 3 (see Table 1) were performed with magnets of different sizes, 3 mm and 5 mm, and similar magnetization (1.32–1.37 T and 1.29–1.32 T, respectively).

**Figure 6:**
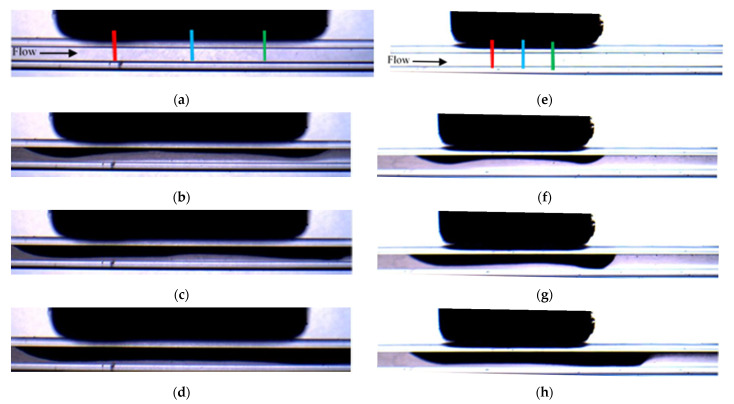
Snapshots of the deposition for experiments PE-M5-P1-C3-QM (**a**–**d**) and PE-M3-P1-C3-QM (**e**–**h**). The non-dimensional time are for (**a**,**e**) $t{\;}^{*}$ = 4.47 × 10^1^, for (**b**,**f**) $t{\;}^{*}$ = 4.47 × 10^3^, for (**c**,**g**) $t{\;}^{*}$ = 8.95 × 10^3^, and for (**d**,**h**) $t{\;}^{*}$ = 1.79 × 10^4^.

**Figure 7:**
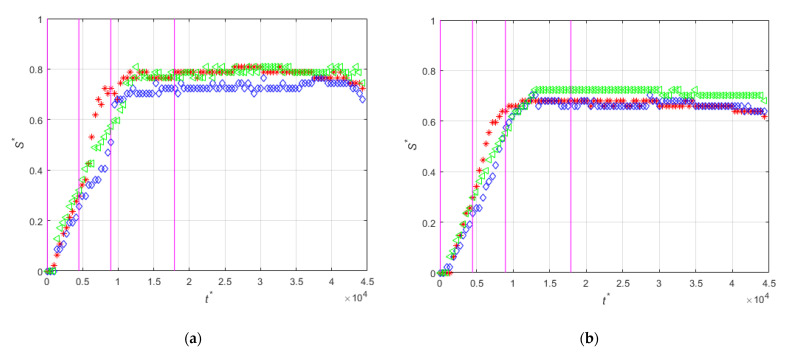
Time-evolutions of the non-dimensional thickness of the magnetic particle deposits at positions ${z^{\prime }}_{\frac{1}{4}}$ (red),$\;{z^{\prime }}_{0}$ (blue), and ${z^{\prime }}_{-\frac{1}{4}}$ (green). Medium flow rate (**a**) PE-M5-P1-C3-QM and (**b**) PE-M3-P1-C3-QM. Vertical lines corresponds to times $t{\;}^{*}$ = 4.47 × 10^1^, $t^{\;*}$ = 4.47 × 10^3^, $t{\;}^{*}$ = 8.95 × 10^3^, and $t{\;}^{*}$ = 1.79 × 10^4^ which correspond to the snapshots of Figure 6.

To analyze the effect of magnet size on the particle accumulation, Figure 6 shows the snapshots of the experiments PE-M5-P1-C3-QM and PE-M3-P1-C3-QM, corresponding to the medium flow rate and the perpendicular orientation of the magnetization vector and Figure 7 shows the corresponding time evolutions of the thicknesses. It can be seen that, initially, the shape of the accumulation of particles is similar for both magnets, with a larger extension of the deposition for the larger magnet. For the magnet of 5 mm, the accumulation of particles is somewhat larger near the leading edge than the trailing edge and later tends to be equal and greater than the accumulation near the center. In the case of the experiment PE-M3-P1-C3-QM, the initial accumulations near the leading edge and near the trailing edge are similar. As time evolves it is observed that the accumulations near the leading edge and near the center are very similar (red and blue curves in Figure 7b) while the accumulation of particles near the trailing edge is larger (green curve in Figure 7b).

### 3.3. Effect of the Diameter of the Particles

#### 3.3.1. Magnet Size of 5 mm

**Figure 8:**
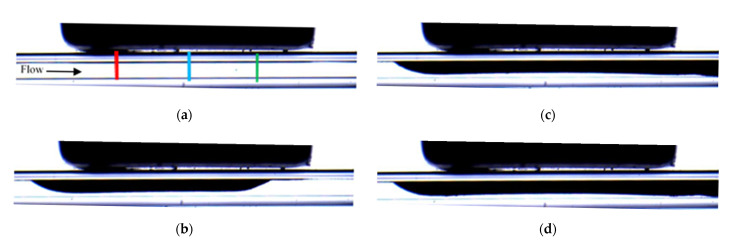
Snapshots of the deposition for the experiment PE-M5-P4-C3-QM at times, (**a**) $t{\;}^{*}$ = 4.47 × 10^1^, (**b**) $t{\;}^{*}$ = 4.47 × 10^3^, (**c**) $t{\;}^{*}$ = 8.95 × 10^3^, (**d**) $t{\;}^{*}$ = 1.79 × 10^4^.

**Figure 9:**
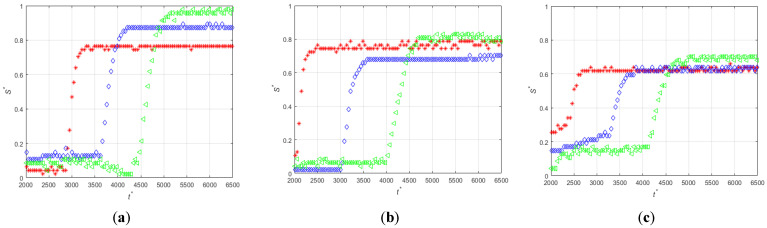
Time-evolutions of the non-dimensional thickness of the magnetic particle deposits at positions ${z^{\prime }}_{\frac{1}{4}}$ (red),$\;{z^{\prime }}_{0}$ (blue) and ${z^{\prime }}_{-\frac{1}{4}}$ (green) for PE-M5-P4-C3, (**a**) low flow rate, (**b**) medium flow rate, and (**c**) high flow rate.

The effect of the mean diameter of the magnetic particles can be observed by comparing the results obtained in experiments for particles with mean diameter 1.14 µm and 4.37 µm, PE-M5-P1-C3 (Set 2) and PE-M5-P4-C3 (Set 4), respectively. As an example, Figure 8 shows the snapshots of the deposition for PE-M5-P4-C3-QM. In this case, the accumulation of particles is performed according to the direction of the flow. First, particles are accumulated near the leading edge of the magnet, then near the center, and finally near the trailing edge and the deposit grows following a moving front along the streamwise direction. This is clearly seen in Figure 9, which shows the time evolutions of the thicknesses of the deposit at the positions indicated in Figure 8a and for the three different flow rates analyzed. Note that, independently of the flow rate, the accumulation of particles starts to increase visibly at $t{\;}^{*}>2000$. This behavior is different from the one observed in the experiment PE-M5-P1-C3 carried out in the same working conditions and shown in Figure 4e–h.

**Figure 10:**
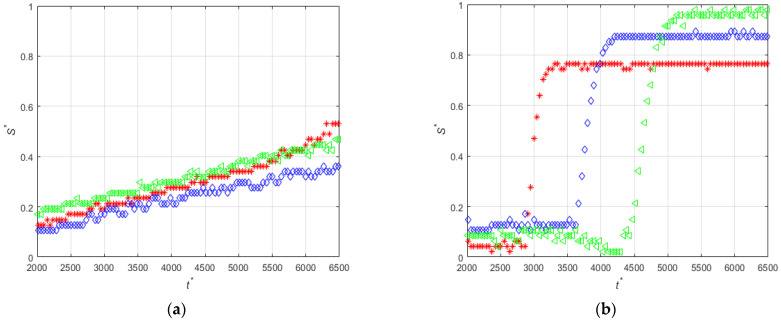
Time-evolutions of the non-dimensional thickness of the magnetic particle deposits at positions ${z^{\prime }}_{\frac{1}{4}}$ (red),$\;{z^{\prime }}_{0}$ (blue), and ${z^{\prime }}_{-\frac{1}{4}}$ (green) for (**a**) PE-M5-P1-C3-QM and (**b**) PE-M5-P4-C3-QM.

To highlight the differences between the experiments PE-M5-P1-C3 and PE-M5-P4-C3 Figure 10 shows the time-evolution of the non-dimensional thickness of the magnetic particles deposits obtained at medium flow rate during the initial times (2000 < $t{\;}^{*}$< 6500). It is clearly observed that the use of particles with an average diameter of 4.37 μm completely modifies the behavior of the accumulation, obtaining the maximum accumulation much faster than in the case of the particles with a diameter of 1.14 μm. For the large particles, the generation of two lobes (Figure 6b) is not observed and the deposit grows following a moving front along the streamwise direction (Figure 8b).

#### 3.3.2. Magnet Size of 3 mm

**Figure 11:**
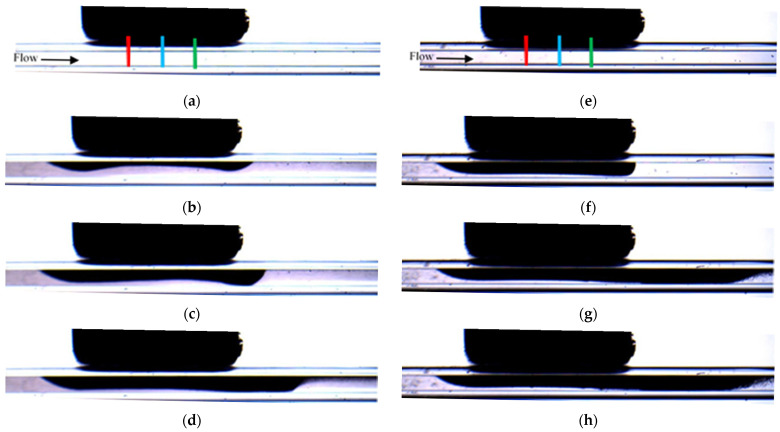
Snapshots of the deposition Particle accumulations for the experiments PE-M3-P1-C3-QM (**a**–**d**) and PE-M3-P4-C3-QM (**e**–**h**). The non-dimensional time are for (**a**,**e**) $t{\;}^{*}$ = 4.47 × 10^1^, for (**b**,**f**) $t{\;}^{*}$ = 4.47 × 10^3^, for (**c**,**g**) $t{\;}^{*}$ = 8.95 × 10^3^ and for (**d**,**h**) $t^{\;*}$ = 1.79 × 10^4^.

As in experiments PE-M5-P1-C3 and PE-M5-P4-C3, the effect of the diameter of the particles can be analyzed using the magnet of 3 mm. Figure 11 shows selected snapshots of the deposition of particles for experiments PE-M3-P1-C3-QM and PE-M3-P4-C3-QM. As in the experiments shown in sub Section 3.2, the deposition of the particles with diameter 1.14 μm shows two lobes near the edges of the magnet, which are symmetrically distributed with respect to the center of the magnet. For the large particles, with a diameter of 4.37 μm, the accumulation grows following a moving front along the streamwise direction. An interesting phenomenon is observed when comparing experiments PE-M3-P1-C3-QM and PE-M3-P4-C3-QM carried out with the small magnet and different particle diameters. It can be seen that for the large particles the accumulation of particles extends far downstream from the trailing edge of the magnet at a distance of the order of the size of the magnet. For the small particles with a diameter of 1.14 μm the downstream extension of the accumulation is approximately half of the size of the magnet. The amount of magnetic material in the particles is about 12% and this different behavior can be attributed to the larger amount of magnetic material for the larger particles. To check if this can be produced by the effect of gravity, we performed an experiment with the microchannel in horizonal orientation, perpendicular to the gravity vector. The visualizations, not shown here, reveal that the shape and extension of the deposition is independent of the orientation of the microchannel.

**Figure 12:**
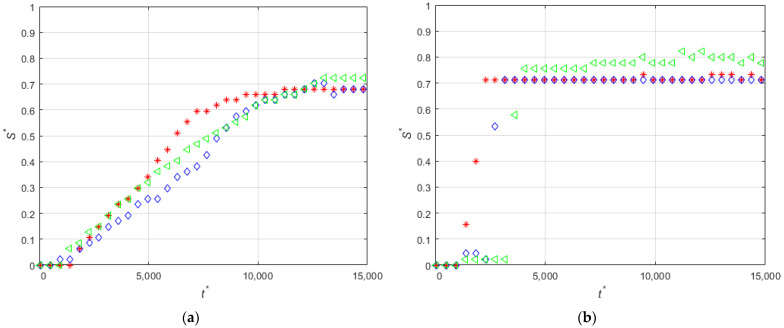
Time-evolutions of the non-dimensional thickness of the magnetic particle deposits at positions ${z^{\prime }}_{\frac{1}{4}}$ (red),$\;{z^{\prime }}_{0}$ (blue), and ${z^{\prime }}_{-\frac{1}{4}}$ (green) for (**a**) PE-M3-P1-C3-QM and (**b**) PE-M3-P4-C3-QM.

Figure 12 compares the time evolution of the non-dimensional thickness for experiments PE-M3-P1-C3-QM and PE-M3-P4-C3-QM carried out with the same conditions but with different particle sizes. It can be seen that large particles are deposited at a significantly larger rate.

### 3.4. Effect of the Hydraulic Diameter of the Microchannel

Experiment PE-M5-P4-C6-QL was carried out with a square microchannel with a hydraulic diameter of 600 µm with a flow rate of 0.03 mL/min. Under these conditions, the average flow velocity is 1.4 mm/s, which is the minimum velocity of all the experiments indicated in Table 1.

**Figure 13:**
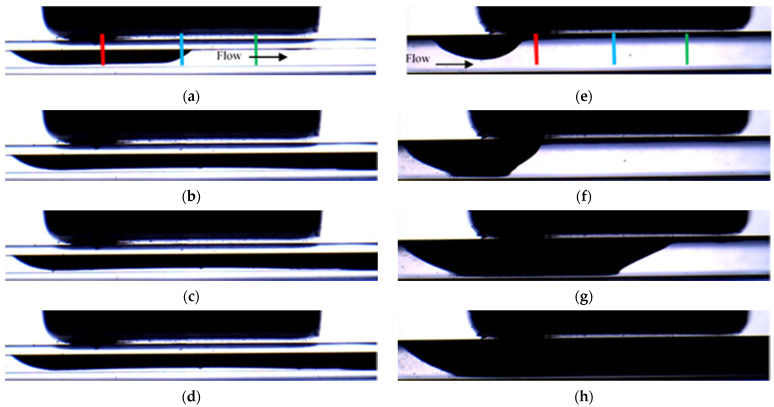
Snapshots of the depositions for PE-M5-P4-C3-QL and PE-M5-P4-C6-QL at different times. For real time t= 200 s ((**a**)$\;t{\;}^{*}$ = 4.47 × 10^3^ and (**e**) $t{\;}^{*}$ = 4.88 × 10^2^), t=400 s ((**b**) $t{\;}^{*}$ = 8.95 × 10^3^, and (**f**) $t{\;}^{*}$ = 9.76 × 10^2^), t=800 s ((**c**) $t^{*}$ = 1.79 × 10^4^ and (**g**) $t{\;}^{*}$ = 1.95 × 10^3^) and t=1000 s ((**d**) $t{\;}^{*}$ = 2.24 × 10^4^ and (**h**) $t{\;}^{*}$ = 2.44 × 10^3^).

**Figure 14:**
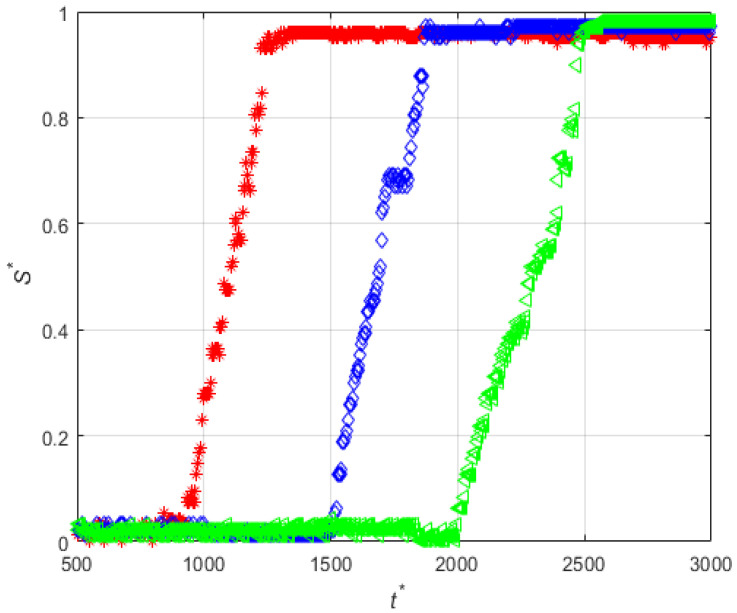
Time-evolutions of the non-dimensional thickness of the magnetic particle deposits at positions ${z^{\prime }}_{\frac{1}{4}}$ (red),$\;{z^{\prime }}_{0}$ (blue), and ${z^{\prime }}_{-\frac{1}{4}}$ (green) for PE-M5-P4-C6-QL.

Figure 13 compares the visualizations of the deposition for experiments PE-M5-P4-C3-QL and PE-M5-P4-C6-QL. It can be seen that for both cases the deposition starts near the leading edge of the magnet. For the microchannel with larger diameter, the deposition extends along the direction perpendicular to the flow and reaches the opposite wall of the microchannel (see Figure 13e). It should be noted that under this condition the flow is not interrupted by the deposit. The fluid, which is pumped continuously by the syringe pump, flows through the central part of the microchannel while the particles are progressively deposited onto the sidewalls of the microchannel perpendicular to the direction of visualization. The deposit corresponding to the experiment with the microchannel of the small diameter (PE-M5-P4-C3-QL, Figure 13a–d) also starts near the leading edge of the magnet and it progressively extends, mainly, along the streamwise direction. The small gap between the deposit and the bottom wall seen in the two-dimensional visualizations of Figure 13a–d suggests that, also in this case, the particles are deposited on the sidewalls of the microchannels, which are perpendicular to the visualization axis. This can be attributed to the relatively large flow velocities attained by the progressive reduction of the available cross-sectional area for the flow as the particle deposit grows locally near the leading edge of the magnet. The comparison of Figure 9a and Figure 14 shows that the particles accumulate first in the leading edge, then in the center and finally on the trailing edge in the case of PE-M5-P4-C6-QL. The biggest difference in the time-evolution of the non-dimensional thickness of magnetic particle deposits between PE-M5-P4-C3-QL and PE-M5-P4-C6-QL is the final value reached. For PE-M5-P4-C6-QL the values reached on the leading edge, central part, and trailing edge are practically the same but for PE-M5-P4-C3-QL the non-dimensional thickness of deposit is higher for the trailing edge and the smallest for the leading edge, having an intermediate value at the center.

### 3.5. Effect of the Concentration of Non-Magnetic Particles on the Deposition of the Magnetic Beads

To study the influence of the non-magnetic particles in the deposition behavior of magnetic particles, four experiments were performed (Set 6 in Table 1) with different concentrations of non-magnetic particles.

**Figure 15:**
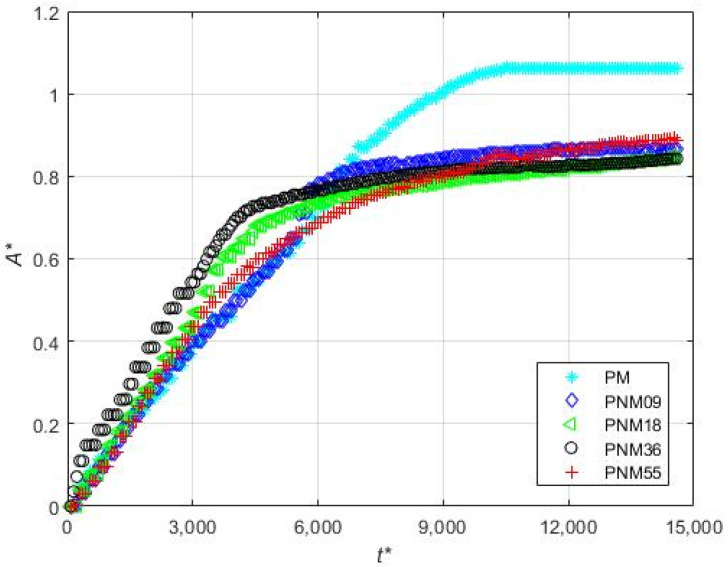
Time-evolutions of the non-dimensional area of the deposited magnetic particles according to Set 6 of Table 1.

Figure 15 shows the time evolution of the non-dimensional total area accumulated ($A{\;}^{*}=A/\left(L_{m}W\right)$ were A is the total calculated area in $m^{2}$) of magnetic particles. It can be seen that time evolution of the area of the deposit corresponding to the experiment without non-magnetic beads reaches a plateau at $t^{*}\approx 10,000$, while the area for the experiments with non-magnetic beads initially increases, in general faster than the experiment with only magnetic particles, and then progressively increases at a much lower rate. Interestingly, the initial rate of growth of the deposit depends on the concentration of non-magnetic particles. It can be seen in Figure 15 that the fastest initial growth is obtained for the experiment PNM36, then for PNM18, then for the largest concentration of non-magnetic particles (PNM55), and finally for the experiment with the lowest concentration (PNM09) which exhibits a similar initial growth rate as the experiment without non-magnetic particles.

**Figure 16:**
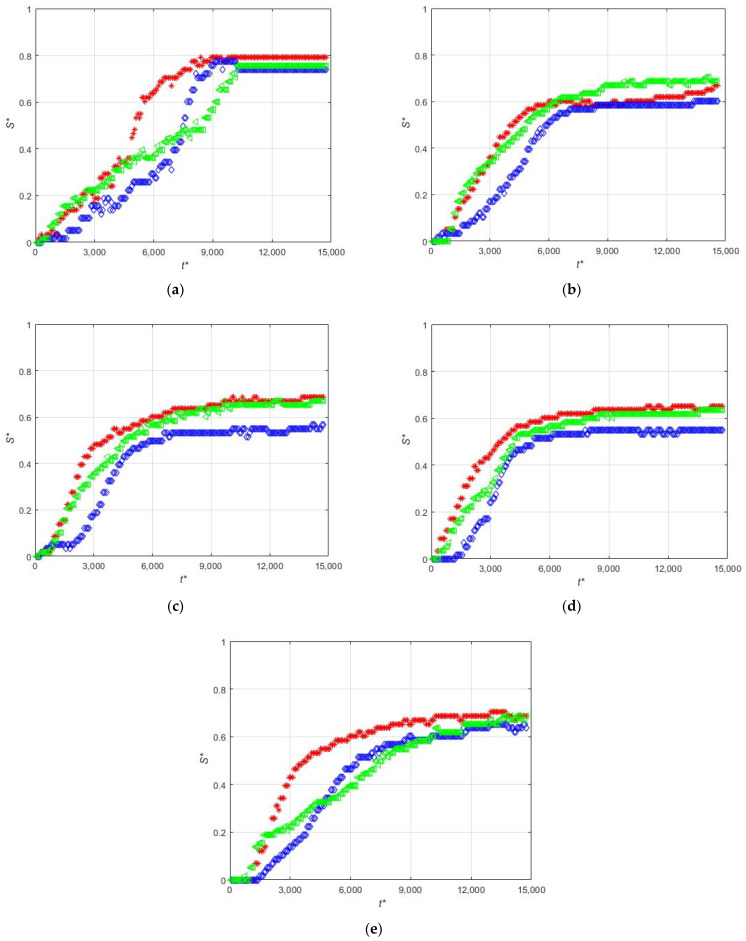
Length accumulation at ${z^{\prime }}_{\frac{1}{4}}$ (red),$\;{z^{\prime }}_{0}$ (blue), and ${z^{\prime }}_{-\frac{1}{4}}$ (green) for experiments (**a**) PE-M5-P1-C3-QM-PM; (**b**) PE-M5-P1-C3-QM-PNM09; (**c**) PE-M5-P1-C3-QM-PNM18; (**d**) PE-M5-P1-C3-QM-PNM36; (**e**) PE-M5-P1-C3-QM-PNM55.

Figure 16 shows the time evolution of the non-dimensional thickness of the magnetic deposit when non-magnetic particles are present in different concentrations. It can be seen that, in general, for all the cases the accumulation near the leading edge of the magnet at the beginning of the magnet is larger than near the center of the magnet. For the experiment with only magnetic beads, the deposition near the trailing edge end is similar to that near the leading edge of the magnet (see Figure 16a). In the rest of the cases with non-magnetic particles, the accumulation in all the positions except for the leading edge (${z^{\prime }}_{\frac{1}{4}}$) is decreased. Figure 16a–c shows that the increase of the concentration of non-magnetic particles produces a relatively fast increase of the deposition near the center of the magnet. This explains the larger accumulation rate for intermediate concentration of non-magnetic particles shown in Figure 15. In this case, for moderate concentrations of non-magnetic particles the shielding effect of the much larger non-magnetic particles on the magnetic beads produces the displacement of the deposition toward the center of the magnet. For large concentrations of non-magnetic particles, the shielding effect decreases the deposition of the magnetic beads.

**Figure 17:**
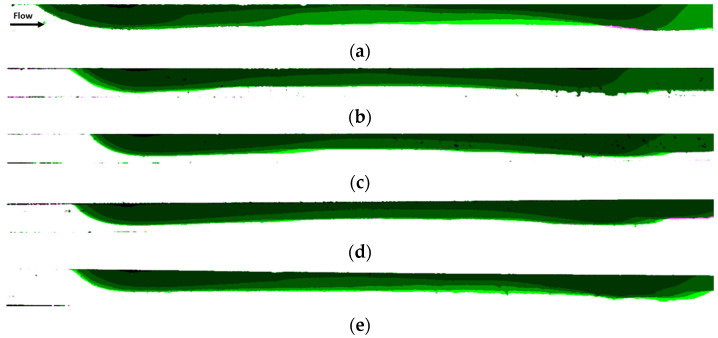
Accumulation of magnetic particles comparison at non-dimensional times $t{\;}^{*}$ = 1, 3000, 6000, 9000, 12,000 for experiments (**a**) PE-M5-P1-C3-QM-PM; (**b**) PE-M5-P1-C3-QM-PNM09; (**c**) PE-M5-P1-C3-QM-PNM18; (**d**) PE-M5-P1-C3-QM-PNM36; (**e**) PE-M5-P1-C3-QM-PNM55.

Figure 17 illustrates the time evolution of the accumulation for different dimensionless times ($t^{*}$ = 1; 3,000; 6,000; 9,000; 12,000) for each experiment. The accumulation is shown in terms of different contours corresponding to different times.

For the experiment with only magnetic particles (Figure 17a) the accumulation near the leading edge of the magnet has a sharper shape than in the experiments with non-magnetic beads. On the other hand, the accumulation in the central zone of the magnet is larger when there are non-magnetic beads in the solution. This can be attributed to the shielding effect of the non-magnetic particles that avoids the deposition near the leading edge of the magnet. In fact, the comparison of Figure 17a,b or Figure 17c shows that the non-magnetic particles promote the early deposition of magnetic beads downstream the trailing edge of the magnet.

## 4. Conclusions

We performed experiments to analyze the spatial distribution and the growth rate of depositions of magnetic particles on the walls of microchannels by the effect of a nearby permanent magnet. Visualizations of the time evolution of the accumulation have been performed under different conditions.

It has been found that the flow rate affects significantly the deposition rate with larger deposition rates at lower flow rates. The direction of the magnetization vector with respect to the flow also has a relevant effect on the spatial distribution and the growth rate of the accumulation. In general, when the magnetization vector is perpendicular to the microchannel wall in contact with the magnet the deposition starts and grows near the leading and trailing edges of the magnet. For orientations of the magnetization vector parallel to the microchannel wall in contact with the magnet the deposition starts near the center of the magnet and at a lower rate according to the weaker spatial distribution of the magnetic field generated by the magnet within the fluid. The size of the magnet is also relevant. Larger magnets with similar magnetization enhance the deposition in comparison with the use of smaller magnets. In addition, larger magnetic particles with a similar concentration of magnetic material generate faster and more localized depositions than smaller magnetic particles. The addition of non-magnetic particles, which have ten times the mean diameter than magnetic particles, decreases the deposition of these in any case. In spite of this fact, it has been observed that solutions with non-magnetic particles accelerate the initial deposition because of the shielding effect of non-magnetic particles. This also produces the displacement of the deposition downstream from the leading edge of the magnet.

## Figures and Tables

**Table 1:** Experimental conditions. Magnetization vector, MS: magnet width, CD: capillary diameter, PD: magnetic particle diameter, and FR: flow rate.

Set	Experiment Label	Magnetization Vector Direction	MS (mm)	CD (μm)	PD (μm)	FR (mL/min)
1	PA-M5-P1-C3-QL	∥	5	300	1.14	0.03
	PA-M5-P1-C3-QM					0.06
	PA-M5-P1-C3-QH					0.12
2	PE-M5-P1-C3-QL	⊥	5	300	1.14	0.03
	PE-M5-P1-C3-QM					0.06
	PE-M5-P1-C3-QH					0.12
3	PE-M3-P1-C3-QM	⊥	3	300	1.14	0.06
	PE-M3-P4-C3-QM				4.37	
4	PE-M5-P4-C3-QL	⊥	5	300	4.37	0.03
	PE-M5-P4-C3-QM					0.06
	PE-M5-P4-C3-QH					0.12
5	PE-M5-P4-C6-QL	⊥	5	600	4.37	0.03
6	PE-M5-P1-C3-QM-PM	⊥	5	300	1.14	0.06
	PE-M5-P1-C3-QM-PNM09					
	PE-M5-P1-C3-QM-PNM18					
	PE-M5-P1-C3-QM-PNM36					
	PE-M5-P1-C3-QM-PNM55					

## Nomenclature

g	[m/s^2^]	Gravity acceleration
L	[m]	Length
S	[m]	Width of the accumulation
W	[m]	Width of the channel
A	[m^2^]	Area of the accumulation
M_s_	[A/m]	Magnetization of the magnet
$\bar{V}$	[m/s]	Mean Velocity
*x*	[m]	Cartesian axis direction
*y*	[m]	Cartesian axis direction
*z*	[m]	Cartesian axis direction
Δt	[s]	Time between images
nframes	[-]	Number of images
**Non Dimensional Numbers**		
$t^{*}$	[-]	$t^{*}=\left(\Delta t\cdot nframes\cdot \bar{V}\right)/W$
$A^{*}$	[-]	$A^{*}=A/\left(L_{m}W\right)$
$S^{*}$	[-]	$S^{*}=S/W$
**Subscripts**		
m		Magnet, magnetic

## References

[1] Dreyfus R., Tabeling P., Willaime H. (2003). Ordered and Disordered Patterns in Two-Phase Flows in Microchannels. Phys. Rev. Lett..

[2] Stone H.A., Stroock A.D., Ajdari A. (2004). Engineering Flows in Small Devices: Microfluidics toward a Lab-on-a-Chip. Annu. Rev. Fluid Mech..

[3] Link D.R., Anna S.L., Weitz D.A., Stone H.A. (2004). Geometrically Mediated Breakup of Drops in Microfluidic Devices. Phys. Rev. Lett..

[4] Beatus T., Tlusty T., Bar-Ziv R. (2006). Phonons in a one-dimensional microfluidic crystal. Nat. Phys..

[5] Beatus T., Bar-Ziv R.H., Tlusty T. (2012). The physics of 2D microfluidic droplet ensembles. Phys. Rep..

[6] Pamme N. (2007). Continuous flow separations in microfluidic devices. Lab Chip.

[7] Bhagat A.A.S., Bow H., Hou H.W., Tan S.J., Han J., Lim C.T. (2010). Microfluidics for cell separation. Med. Biol. Eng. Comput..

[8] Sajeesh P., Sen A.K. (2014). Particle separation and sorting in microfluidic devices: A review. Microfluid. Nanofluid..

[9] Lindner A. (2014). Flow of complex suspensions. Phys. Fluids.

[10] Das S., Garg A., Campbell A.I., Howse J., Sen A., Velegol D., Golestanian R., Ebbens S.J. (2015). Boundaries can steer active Janus spheres. Nat. Commun..

[11] Bechinger C., Di Leonardo R., Löwen H., Reichhardt C., Volpe G., Volpe G. (2016). Active particles in complex and crowded environments. Rev. Mod. Phys..

[12] Yan J., Bloom M., Bae S.C., Luijten E., Granick S. (2012). Linking synchronization to self-assembly using magnetic Janus colloids. Nature.

[13] Hejazian M., Li W., Nguyen N.-T. (2015). Lab on a chip for continuous-flow magnetic cell separation. Lab Chip.

[14] Huang W., Yang F., Zhu L., Qiao R., Zhao Y. (2017). Manipulation of magnetic nanorod clusters in liquid by non-uniform alternating magnetic fields. Soft Matter.

[15] Peyer K.E., Zhang L., Nelson B.J. (2013). Bio-inspired magnetic swimming microrobots for biomedical applications. Nanoscale.

[16] Hamilton J.K., Petrov P.G., Winlove C.P., Gilbert A.D., Bryan M.T., Ogrin F.Y. (2017). Magnetically controlled ferromagnetic swimmers. Sci. Rep..

[17] Pallares J. (2016). A Criterion for the Complete Deposition of Magnetic Beads on the Walls of Microchannels. PLoS ONE.

[18] Zhou R., Bai F., Wang C. (2017). Magnetic separation of microparticles by shape. Lab Chip.

[19] Lau I.P., Chen H., Wang J., Ong H.C., Leung K.C.-F., Ho H.P., Kong S.K. (2012). In vitro effect of CTAB- and PEG-coated gold nanorods on the induction of eryptosis/erythroptosis in human erythrocytes. Nanotoxicology.

[20] Wilson M.R., Lightbody J.H., Donaldson K., Sales J., Stone V. (2002). Interactions between ultrafine particles and transition metals in vivo and in vitro. Toxicol. Appl. Pharmacol..

[21] Schwarze P.E., Øvrevik J., Hetland R.B., Becher R., Cassee F.R., Låg M., Løvik M., Dybing E., Refsnes M. (2007). Importance of Size and Composition of Particles for Effects on Cells In Vitro. Inhal. Toxicol..

[22] Gijs M.A.M., Lacharme F., Lehmann U. (2010). Microfluidic Applications of Magnetic Particles for Biological Analysis and Catalysis. Chem. Rev..

[23] Rosi N.L., Mirkin C.A. (2005). Nanostructures in Biodiagnostics. Chem. Rev..

[24] Santiago A.M., Ribeiro T., Rodrigues A.S., Ribeiro B., Frade R.F.M., Baleizão C., Farinha J.P.S. (2015). Multifunctional Hybrid Silica Nanoparticles with a Fluorescent Core and Active Targeting Shell for Fluorescence Imaging Biodiagnostic Applications. Eur. J. Inorg. Chem..

[25] Pissuwan D., Valenzuela S.M., Cortie M.B. (2008). Prospects for Gold Nanorod Particles in Diagnostic and Therapeutic Applications. Biotechnol. Genet. Eng. Rev..

[26] Chandra P., Singh J., Singh A., Srivastava A., Goyal R.N., Shim Y.B. (2013). Gold Nanoparticles and Nanocomposites in Clinical Diagnostics Using Electrochemical Methods. J. Nanopart..

[27] Almomani T., Udaykumar H.S., Marshall J.S., Chandran K.B. (2008). Micro-scale Dynamic Simulation of Erythrocyte–Platelet Interaction in Blood Flow. Ann. Biomed. Eng..

[28] Chen D., Xi T., Bai J. (2007). Biological effects induced by nanosilver particles: In vivo study. Biomed. Mater..

[29] Güunter O. (2000). Toxicology of ultrafine particles: In vivo studies. Phil. Trans. R. Soc. A.

[30] Castro J.R., Saunders W.M., Tobias C.A., Chen G.T.Y., Curtis S., Lyman J.T., Collier J.M., Pitluck S., Woodruff K.A., Blakely E.A. (1982). Treatment of cancer with heavy charged particles. Int. J. Radiat. Oncol. Biol. Phys..

[31] Nemmar A., Hoylaerts M.F., Hoet P.H., Dinsdale D., Smith T., Xu H., Vermylen J., Nemery B. (2002). Ultrafine particles affect experimental thrombosis in an in vivo hamster model. Am. J. Respir. Crit. Care Med..

[32] Rosendaal F.R. (1999). Venous thrombosis: A multicausal disease. Lancet.

[33] Kyrle P.A., Eichinger S. (2005). Deep vein thrombosis. Lancet.

[34] http://www.spherotech.com/.

[35] https://www.dantecdynamics.com/.

[36] https://www.supermagnete.es/eng/data_table.php.

[37] https://www.cmscientific.eu/.

[38] Shevkoplyas S.S., Siegel A.C., Westervelt R.M., Prentiss M.G., Whitesides G.M. (2007). The force acting on a superparamagnetic bead due to an applied magnetic field. Lab Chip.

